# Potential Use of Carob (*Ceratonia siliqua* L.) Biopolymers in Edible Food Packaging

**DOI:** 10.3390/polym18192386

**Published:** 2026-09-30

**Authors:** Osman Onur KARA

**Affiliations:** Ministry of Agriculture and Forestry, Antalya Provincial Directorate, Antalya 07010, Türkiye; osmanonurkara@gmail.com

**Keywords:** carob pod, locust bean gum, edible packaging, pectin, cellulose

## Abstract

The increasing interest in environmentally friendly, biodegradable, and edible packaging materials today has brought the use of agricultural-derived functional biopolymers to the forefront. The carob (*Ceratonia siliqua* L.) pod, with its rich biopolymer components such as carob gum (LBG) in its seeds and pectin and cellulose in its pulp, has high potential in the development of edible packaging matrices. Although these biopolymers exhibit limited water vapor barrier capacity due to their hydrophilic nature, they provide moderate to high barrier properties against oxygen and carbon dioxide. Synergistic effects obtained with different polymer combinations in packaging production can strengthen barrier and durability properties, and the inclusion of plasticizers such as polyethylene glycol and sorbitol in the packaging matrix can improve flexibility, homogeneity, and processability. Furthermore, the inclusion of antimicrobial, antioxidant, and nutritional components in the matrix can impart active functions to the packaging. Carob is of strategic importance in agriculture due to its adaptation to arid climates, its efficient use of water resources thanks to its deep root system, and its erosion-preventing properties. Consequently, the utilization of carob pod and its by-products in edible packaging applications has the potential to significantly contribute to reducing the use of synthetic plastics and promoting ecological and agricultural sustainability.

## 1. Introduction

Foods are packaged to protect them from spoilage microorganisms; reduce the effects of external factors such as oxygen, moisture, and light; ensure storage and transport safety; and inform the consumer about the product content [1,2]. Therefore, food packaging is of critical importance in terms of ensuring the post-processing preservation of both processed and unprocessed products, as well as maintaining their physicochemical and microbiological stability throughout their shelf life [3]. Currently, conventional materials such as plastic, glass, metal, and paper are predominantly used in food packaging [4]. In particular, petroleum-based synthetic plastics such as polyethylene, polypropylene, polystyrene, and polyvinyl chloride are the most widely used packaging materials due to their advantages such as durability, moisture and gas barrier properties, and cost-effectiveness. However, these materials take a very long time to decompose in nature and are difficult to recycle. The increase in packaging use, parallel to the increase in the world population and developments in food technologies, brings with it significant environmental and health risks in terms of the natural cycle [1,5,6]. World plastic production increased from 1.5 million tons in 1950 to 430.9 million tons in 2024, and it has been reported that only 7% of the plastic produced annually can be recycled [5,7]. This situation necessitates the development of environmentally friendly alternatives to petroleum-based plastic packaging. In this context, edible films and coatings produced from biodegradable and food-compatible compounds stand out as promising alternative packaging materials. These materials have the potential to offer additional health benefits to the consumer as they can be consumed with food. In addition, their biocompatibility ensures safety regarding food-packaging interactions [2,5,8,9]. In this context, carob (*Ceratonia siliqua* L.) has strong potential as a sustainable and versatile source, offering distinct biopolymers from different fruit fractions, including locust bean gum from seeds and pectin and cellulose from pulp.

## 2. Literature Search Strategy

A narrative literature search was conducted to identify publications on carob-derived polymers including locust bean gum, pectin, and cellulose for food packaging applications. Searches were performed across Web of Science, Scopus, and Science Direct databases from inception through August 2026. The search combined the terms “carob”, “carob pod”, “carob pulp”, “carob seed”, “locust bean gum”, “LBG”, “pectin”, “cellulose”, “biodegradable food packaging”, “edible film”, “edible coating”, “active packaging”, and “food packaging material” using Boolean operators, e.g., (“carob” OR “carob pod” OR “carob pulp” OR “carob seed”) AND (“locust bean gum” OR “LBG” OR “pectin” OR “cellulose”) AND (“biodegradable food packaging” OR “edible film” OR “edible coating” OR “active packaging” OR “food packaging material”). The reference lists of relevant reviews and research articles were also screened to identify additional publications.

To enhance search specificity and effectively manage the broad body of literature on carob and also bio-based packaging, a structured two-tiered screening logic was applied. In the primary screening phase, titles and abstracts were filtered to exclude studies focusing solely on agricultural production, agronomic traits, or non-packaging applications (e.g., animal feed, clinical pharmacology, or biofuel production). In the secondary screening phase, full-text articles were evaluated based on their explicit focus on matrix development, biopolymer extraction tailored for packaging functions, or functional property characterization.

Priority was given to studies focusing on biodegradable and edible food packaging applications, material properties, and performance evaluation, including foundational studies describing key properties of carob-derived polymers. As this review is narrative, study selection and evidence synthesis were performed qualitatively.

## 3. Edible Packaging

**Figure 1:**
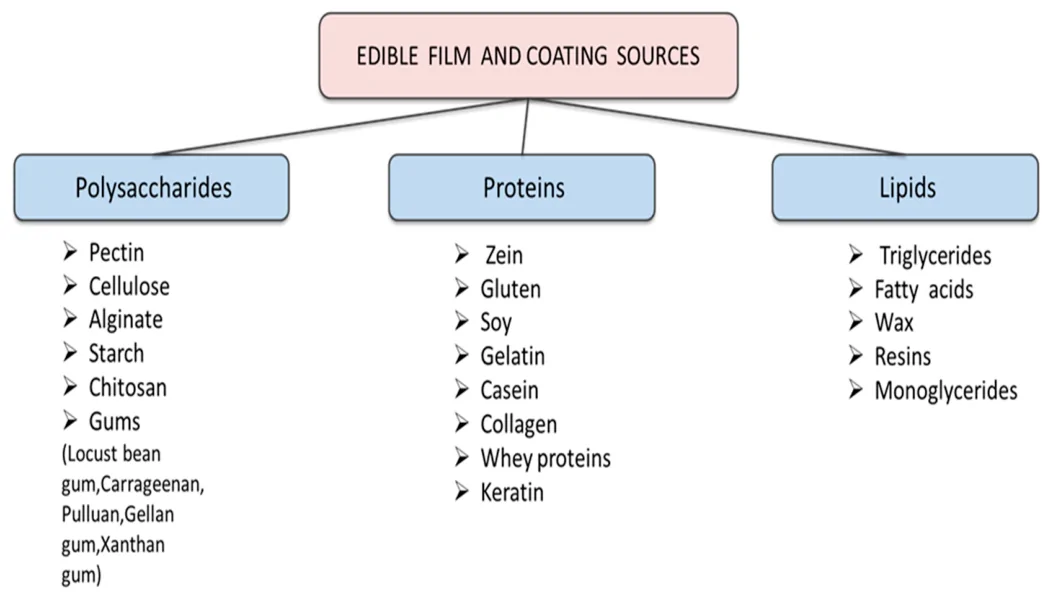
Edible film and coating sources [15,16,17].

The main motivations for using edible food packaging are the increasing consumer demand for safe, healthy and stable foods and the growing awareness of the harmful environmental effects caused by the long-term persistence of non-biodegradable packaging waste in nature [10]. However, the main disadvantages of edible packaging are high production costs, limited mechanical strength, and high moisture permeability [4]. In the production of edible food packaging from biological materials, these materials are converted into edible films and coatings. While films can be utilized in the form of packaging and pouches, coatings are applied directly onto the surface of food products by dipping or spraying methods [1]. In the production of these films and coatings, mainly biopolymers based on carbohydrates and proteins, as well as lipid-based compounds, can be used individually or in combination [11,12]. To evaluate the utilized biopolymers as edible packaging, characteristics such as biodegradability, moisture and oxygen permeability, edibility, aesthetic appearance, prevention of aroma, odor, and lipid migration, toxicological safety, mechanical strength, and cost-efficiency are taken into consideration [13,14]. The most common components used as edible films are shown in Figure 1.

In addition to being eco-friendly and having the potential to reduce chemical hazards and domestic waste, biopolymer-based edible packaging can also be used as functional packaging materials when enriched with various compounds such as antimicrobials, antioxidants, anti-browning agents, and colorants. The controlled release of these compounds from the packaging matrix ensures that their efficacy is maintained over an extended period [2,6,10,18].

The use of edible films and coatings in the food industry is not a new practice. The origins of this practice date back to the 12th century, when wax coatings were used to prevent water loss in fruits. It is known that wax-based coatings were applied to citrus fruits in China during the 12th century. In the 15th century, an edible film made from boiled soy milk, known as “Yuba”, was used in Japan to preserve food quality and improve appearance [10,15,19]. Furthermore, lipid-based coatings have been used on meat and cheese products since the Middle Ages to provide structural protection [16]. Examples of commonly used edible packaging applications today include wax coating of citrus fruits and apples, the use of edible collagen casings in sausages, zein coatings in confectionery, sugar coatings on nuts, and the use of hydroxymethylcellulose films as pouches for some dried foods [4,16].

Biopolymers with protein and polysaccharide structures exhibit better mechanical properties and lower gas permeability to oxygen and carbon dioxide compared to hydrophobic substances. However, due to their hydrophilic structure, their water vapor permeability is high. This disadvantage can be compensated for by combining these biopolymers with hydrophobic components such as lipids and waxes [15]. As a model edible packaging material, soy protein isolate used alone or in combination with corn zein exhibited significantly superior oxygen barrier properties compared to conventional nylon-based low-density polyethylene-based packaging. Specifically, the oxygen permeability of soy protein isolate film (0.60 × 10^−18^ m^3^ m/m^2^ s Pa) and corn zein/soy protein isolate bilayer film (0.81 × 10^−18^ m^3^ m/m^2^ s Pa) was 5.8 and 4.3 times lower than that of nylon-based low-density polyethylene film (3.51 × 10^−18^ m^3^ m/m^2^ s Pa), respectively. However, the same edible films showed considerably higher water vapor permeability, with WVP values of 0.94 × 10^−12^ and 0.61 × 10^−12^ kg m/m^2^ s Pa for soy protein isolate and corn zein/soy protein isolate, respectively, compared to <0.001 × 10^−12^ kg m/m^2^ s Pa for conventional nylon-based low-density polyethylene film [20]. Lipids are also materials used in the production of edible packaging; however, they are not included in the biopolymer class. Lipid compounds such as beeswax, carnauba wax, paraffin wax, and fatty acids can be added to the film solution to impart hydrophobic properties to protein and carbohydrate-based hydrophilic films and coatings, or they can be used alone as surface barriers [16,21,22]. Plasticizers are used in the production of edible packaging to increase the flexibility and improve the mechanical properties of biopolymers. Plasticizers increase molecular mobility by entering between the polymer chains, thus improving the flexibility and other mechanical properties of the material. Thanks to their hygroscopic structure, they can soften the product, preventing problems such as breakage and cracking. For this purpose, polyol-type plasticizers such as glycerol, sorbitol and polyethylene glycol (PEG) are widely used in edible packaging formulations [10,16,21]. In evaluating the structural strength of edible biopolymer packaging, mechanical tests such as tensile strength, elongation at break, elastic modulus, water vapor permeability (WVP), oxygen transmission rate (OTR), compressive strength, hardness, tear strength, burst strength, abrasion resistance, adhesion force and folding strength are used [16,17]. When selecting edible food packaging, the packaging material, the type of food to be used and the application method should be taken into consideration [2]. A comparison of the mechanical and barrier properties of conventional LDPE and biodegradable polymer-based films developed for food packaging applications is presented in Table 1. Conventional LDPE exhibits high elongation at break (121.39%) and a high oxygen transmission rate (353 cc·m^2^/day), while its tensile strength (6.13 MPa) is comparable to that of native corn starch film (6.00 MPa). The incorporation of polysaccharides, locust bean gum and sodium alginate (7.52 MPa) and particularly the addition of blackseed oil to the caseinate-carboxymethyl chitosan matrix (12.5 MPa) resulted in a significant improvement in tensile strength. In terms of flexibility, all biodegradable films showed lower elongation values (8.9–90.5%) compared to LDPE, which is expected due to their more rigid biopolymer network. Regarding barrier properties, native corn starch and locust bean gum/alginate films showed higher WVP values (6.00 and 11.20 g·mm/m^2^·h·kPa), whereas the blackseed oil-supplemented film exhibited the lowest WVP (0.56 g·mm/m^2^·h·kPa) among the biodegradable films, approaching the value of LDPE (0.58 g·mm/m^2^·h·kPa), which can be attributed to the hydrophobic effect of the oil phase as reported in the original study. In terms of oxygen barrier, the OTR of starch-based films (4 cc·m^2^/day) was markedly lower than that of LDPE (353 cc·m^2^/day), confirming their superior oxygen barrier capacity. It should be noted that the data summarized in Table 1 are compiled directly from the respective literature sources as reported by the original authors, without any recalculation or reinterpretation beyond unit standardization to g·mm/m^2^·h·kPa where necessary. The values were obtained under different experimental conditions and are presented here for indicative comparative purposes only [23,24,25,26].

## 4. Carob Pod

**Figure 2:**
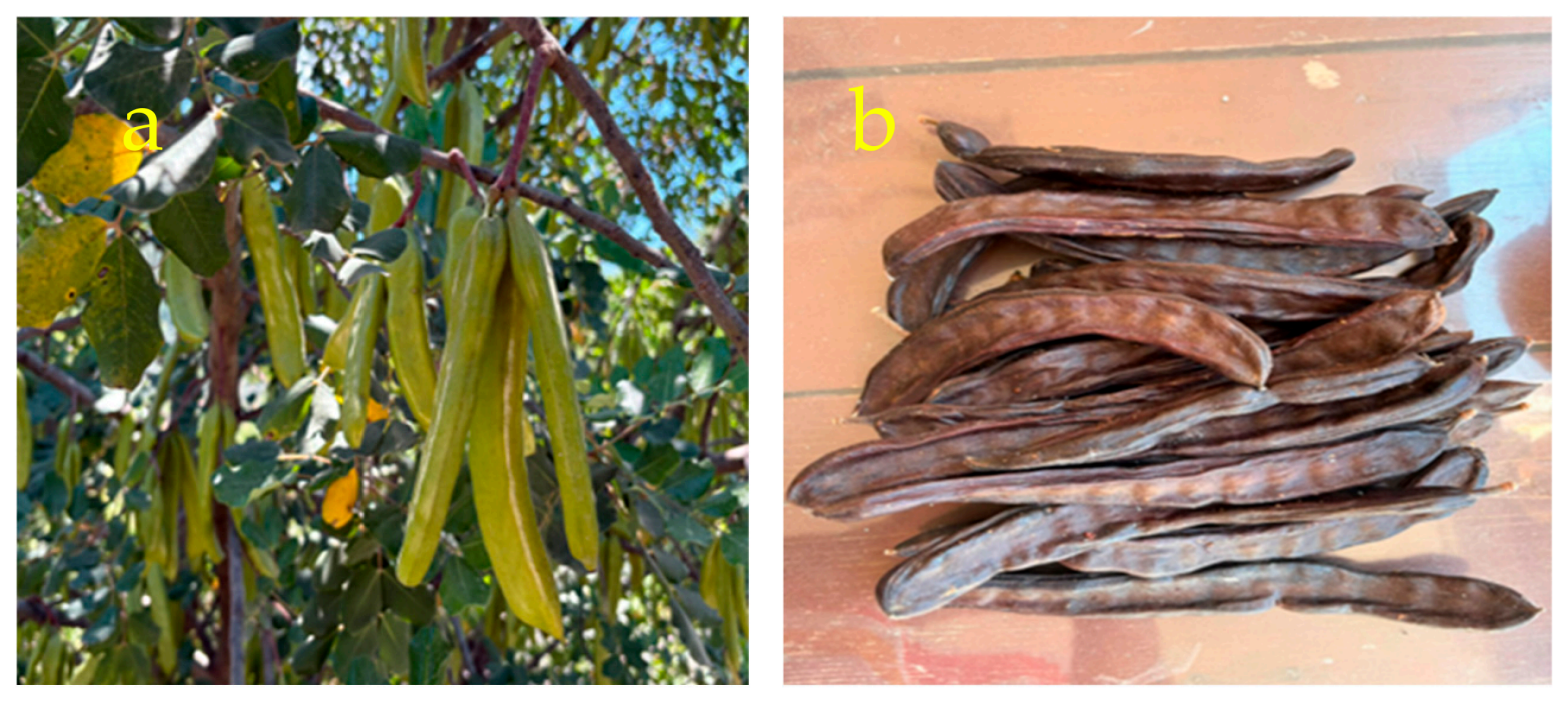

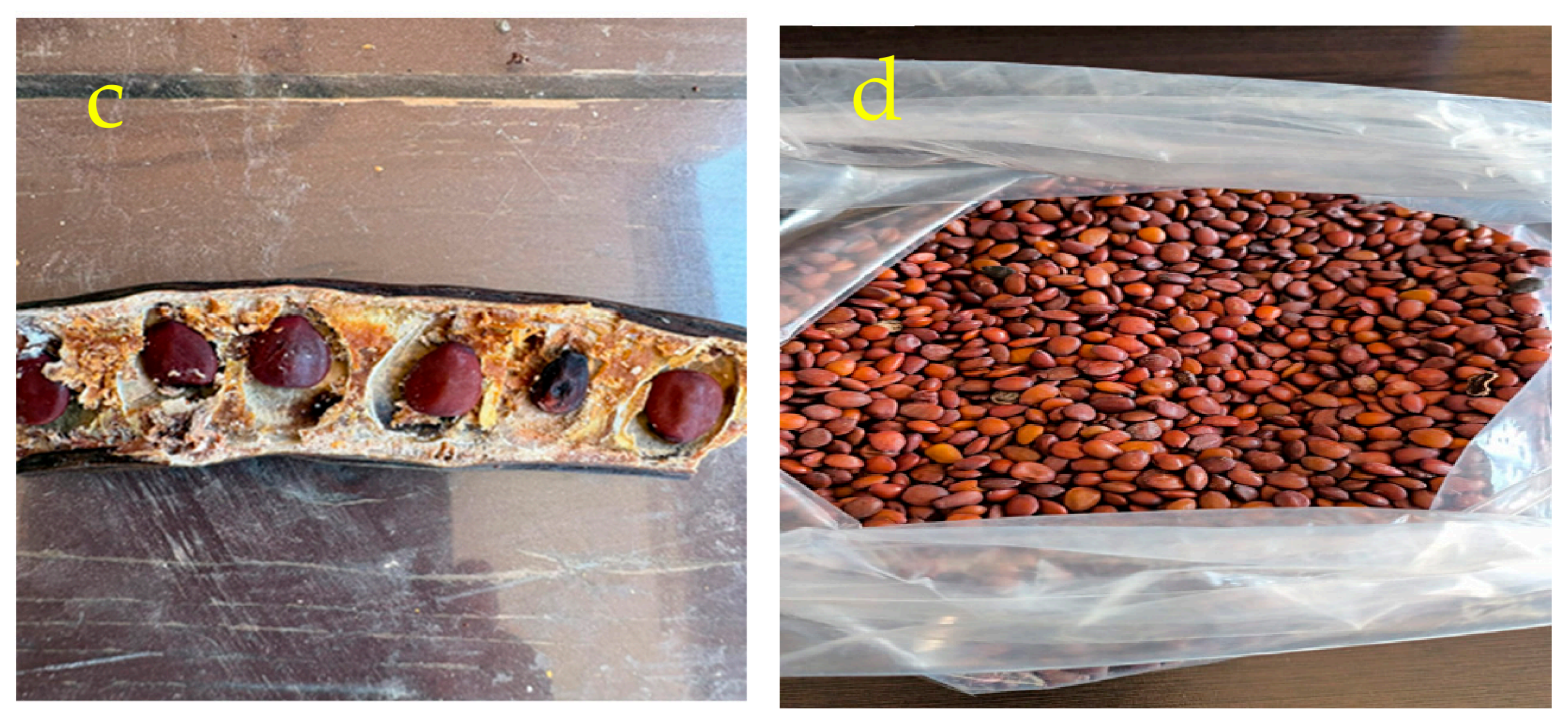
(**a**) Raw carob pod. (**b**) Ripe carob pod. (**c**) Location of seed inside the pod. (**d**) Carob seeds.

Carob (*Ceratonia siliqua* L.) is a long-lived tree that is mainly cultivated around the Mediterranean, and it can reach a height of 8–15 m. Having high resistance to harsh conditions such as drought, carob can grow even in soils unsuitable for agriculture. Thanks to its deep root system, it uses water more efficiently and prevents erosion by holding the soil. Because of this feature, it is considered an ecologically important species. The tree bears fruit known for its nutritional value and economic importance. The fruit of Carob is a legume pod with a straight or slightly curved shape, approximately 10–30 cm long, 1.5–3.5 cm wide and 1 cm thick [27,28,29,30] [Figure 2]. Carob pod has been used for human consumption and animal feed in the Mediterranean Basin from the past to the present [29].

The main carob-producing countries in the world are Spain, Italy, Portugal, Morocco, Turkey, and Greece. According to 2017 data, it is estimated that approximately 174,010 tons of carob pods were produced in an area of about 76.458 hectares worldwide [31,32]. Compared to previous decades, a downward trend in pod production has been observed in the last decade [31]. This situation is thought to be due to the increasing use of alternatives such as guar gum and xanthan gum instead of locust bean gum (LBG, E410), which is obtained from carob seeds and is the main commercial product of carob pods worldwide [30,33].

The carob pod stands out in the food industry with its characteristic strong aroma and high sugar content. It contains 45–52% sugar, up to 40% crude fiber, 2–7% protein, and 1–5% mineral matter [30]. Approximately 90% of the carob pod consists of pulp, while 10% is composed of 10–15 seeds arranged in a row within the pod. Since the chemical compositions of the seeds and pulp differ, they are used separately in most products. The pulp is rich in sugar (48–56%). It is also rich in insoluble fibers such as cellulose and hemicellulose. The dietary fiber content of the pulp is 18%, protein content is 1–2%, and fat content is 0.2–0.6% [34,35]. This part of the pod, which has a high sugar and dietary fiber content, is used in the production of syrup and molasses or ground into flour. Because its aroma profile resembles cocoa when roasted, this flour is used as a cocoa substitute in baked goods and ice cream [33,36]. The most valuable part of the pod is its seeds. Locust bean gum, which has a wide range of applications as a food additive, is produced from the seeds and used as a thickener, stabilizer, and film-forming agent [34,35]. The brown, quite hard seeds weigh approximately 0.2 g. The seed consists of 30–35% hull, 40–50% endosperm, and 20–25% embryo [30,37]. The seed hull contains approximately 75–93% carbohydrate, 3.71–4.86% protein, and 0.31–0.54% fat. The embryo, the essential part of the seed that forms the new plant, consists mainly of 53.13–67.1% protein and 2.26% fat. The endosperm, the most industrially important part of the seed, is generally known as locust bean gum (LBG). It contains 80–85% galactomannan, 5% protein, 1% crude fiber, and 0.5% fat [36,38].

One of the increasingly popular uses of carob pod in recent years is the utilization of its biopolymers in the production of edible food packaging. Locust bean gum, cellulose, and pectin found in the pod offer significant potential for developing eco-friendly and sustainable packaging materials. This study investigates the potential applications of carob pod in the production of edible food packaging. To the best of our knowledge, this is the first study that provides an integrated study of carob-derived biopolymers including LBG, pectin, and cellulose specifically for edible food packaging applications. Evaluating carob biopolymers within a single review is crucial to provide a comprehensive, comparative perspective on how various fractions of the carob biomass can be harnessed for packaging production. While experimental data on the use of LBG in edible films and coatings are well established, studies on carob-derived pectin and cellulose for food packaging applications remain limited. Therefore, this study aims to guide future studies toward the valorization of carob pod for eco-friendly and sustainable food packaging materials.

## 5. Use of Locust Bean Gum (LBG) in Edible Packaging Production

**Figure 3:**
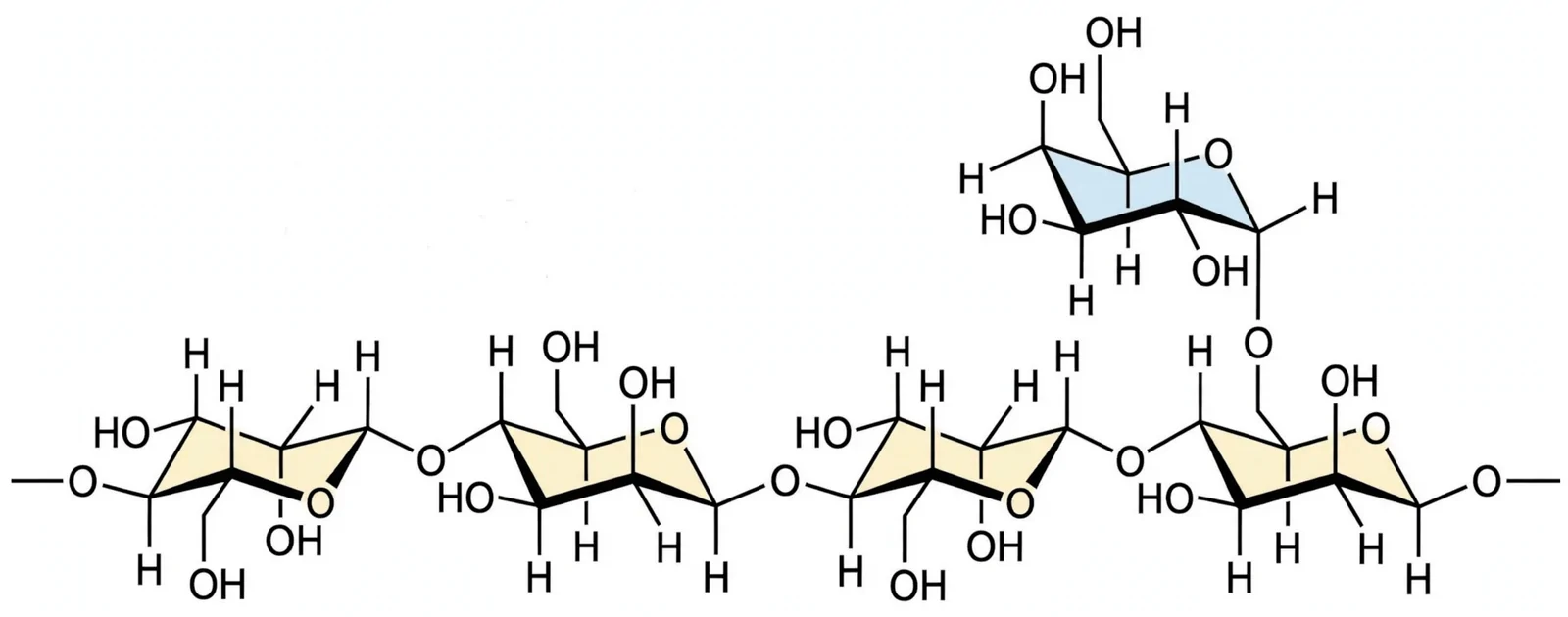
Schematic representation of the structure of locust bean gum.

LBG, extracted from the seed endosperm of the carob pod, is a galactomannan-type gum composed of mannose and galactose monosaccharide units. It is a polysaccharide biopolymer widely utilized across various industrial sectors, including food, chemical, pharmaceutical, textile, paper, and cosmetics. As LBG is a non-ionic polysaccharide, its viscosity and solubility are not influenced by the pH of the liquid medium. Due to its ability to form hydrogen bonds with water molecules, it is primarily used as a thickener, stabilizer, and emulsifier in the food industry [26,38,39]. Chemically, LBG is a branched polymer consisting of a main chain of β-(1 → 4)-D-mannopyranosyl units with α-D-galactopyranosyl single-unit side chains. It is classified as a galactomannan due to the attachment of these galactose side units to the mannan backbone [40,41,42] (Figure 3). LBG is one of three galactomannans of commercial importance in industry, the others being guar gum (mannose-to-galactose (M/G) ratio: 2:1) and tara gum (M/G ratio: 3:1) [43]. The mannose-to-galactose (M/G) ratio directly influences the physicochemical properties of galactomannans, such as solubility and viscosity. This stems from the presence of galactose side groups, which impede intermolecular interactions between galactomannan chains. Consequently, guar gum, possessing a lower M/G ratio, is soluble in cold water, whereas locust bean gum (LBG) is only partially soluble at room temperature. Complete dissolution, optimal viscosity, and maximum water-binding capacity of LBG require thermal treatment [44,45].

The biocompatibility, biodegradability, and film-forming ability of LBG, along with its strong barrier properties against oxygen and carbon dioxide, render it a promising biopolymer for edible packaging films and coating applications [11,40,43,46,47]. Consequently, these attributes establish LBG as a highly suitable polymer for food packaging materials.

### 5.1. LBG Production

**Figure 4:**
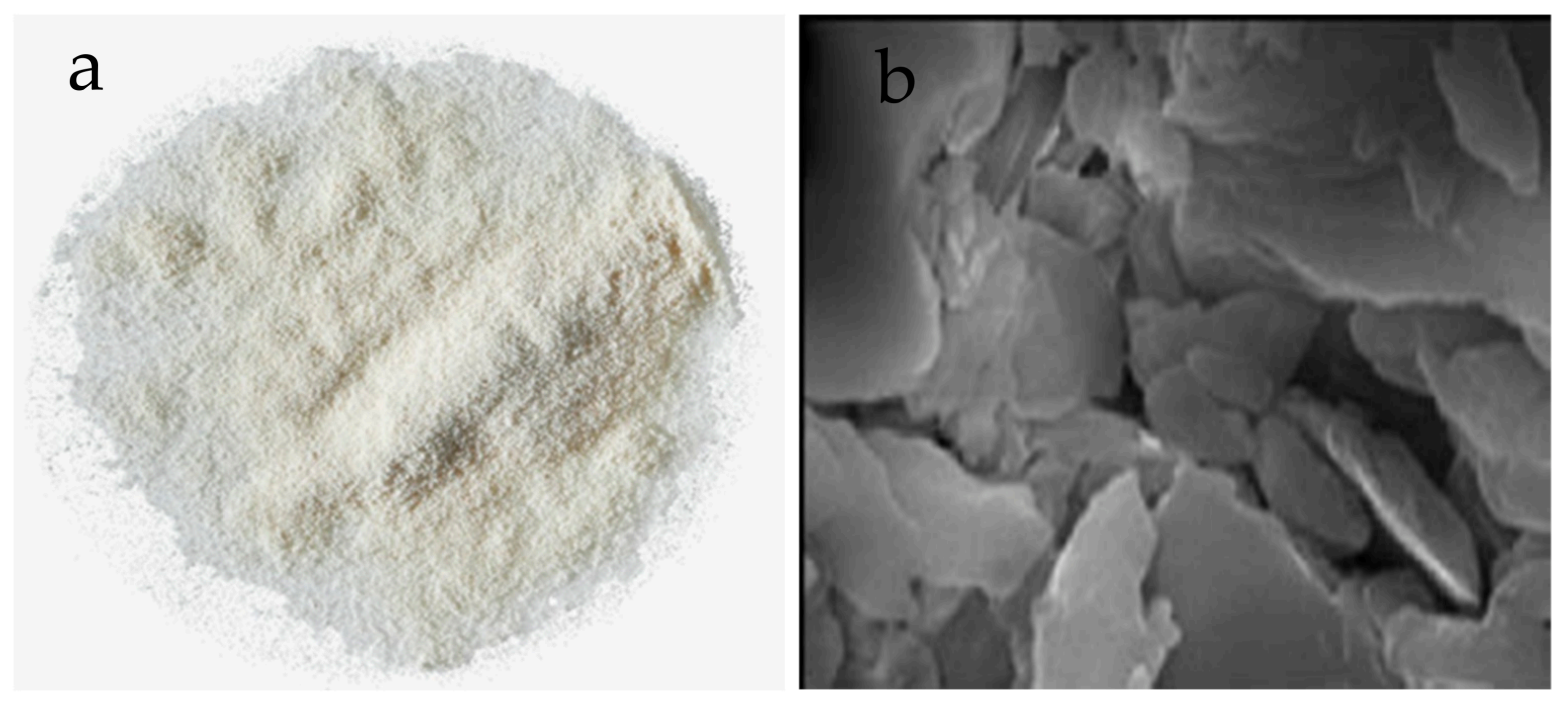
(**a**) Locust bean gum [57]. (**b**) Scanning electron microscope image of LBG [58].

In LBG production, carob seeds are first separated from the pulp. The extremely hard hulls are removed typically using thermal or acid peeling methods. The seeds are then mechanically split, and the inner embryo is separated from the endosperm through a combination of crushing, sieving, and air classification, taking advantage of the differences in their density and friability. The isolated endosperm is mechanically ground to obtain native LBG powder. To clarify and purify the resulting native LBG powder, it is dissolved in water with the aid of heat. This process is carried out at temperatures ranging from 80 °C to 120 °C and after that the solution is subjected to filtration. Then, isopropanol or ethanol is used for the precipitation process. Alcohol is added to achieve a water-to-alcohol ratio between 1:1 and 1:3, after which a second filtration is executed. Finally, drying and re-grinding processes are carried out to obtain a purified, whitish cream-colored LBG powder (Figure 4) [38,43].

Traditionally, acid and thermomechanical roasting processes are used to dehull carob seeds [38,48]. Beyond these traditional processes, more efficient strategies such as hot water extraction and optimization via response surface methodology have been investigated to increase the recovery yield of LBG [48,49]. As eco-friendly alternatives to traditional extraction, innovative technologies such as subcritical water extraction, ultrasound-assisted extraction, microwave-assisted extraction, supercritical fluid extraction, and enzyme-assisted extraction stand out in the extraction of natural products [50,51,52,53]. Although these technologies have not yet been widely applied for LBG extraction, they are considered sustainable green alternatives because they eliminate or reduce use of organic solvents, shorten processing times, and increase process efficiency [54]. In particular, ultrasound-assisted extraction enhances mass transfer through cavitation effects [51], while subcritical water extraction stands out as an effective and environmentally friendly method using only water [50]. In this regard, investigating green and non-conventional technologies in LBG extraction is of great importance in terms of shortening processing times, minimizing chemical use, and preserving polymer quality. The development of these innovative approaches will both reduce environmental impact and pave the way for more sustainable and efficient processes on an industrial scale.

### 5.2. LBG-Based Films and Coatings

**Figure 5:**
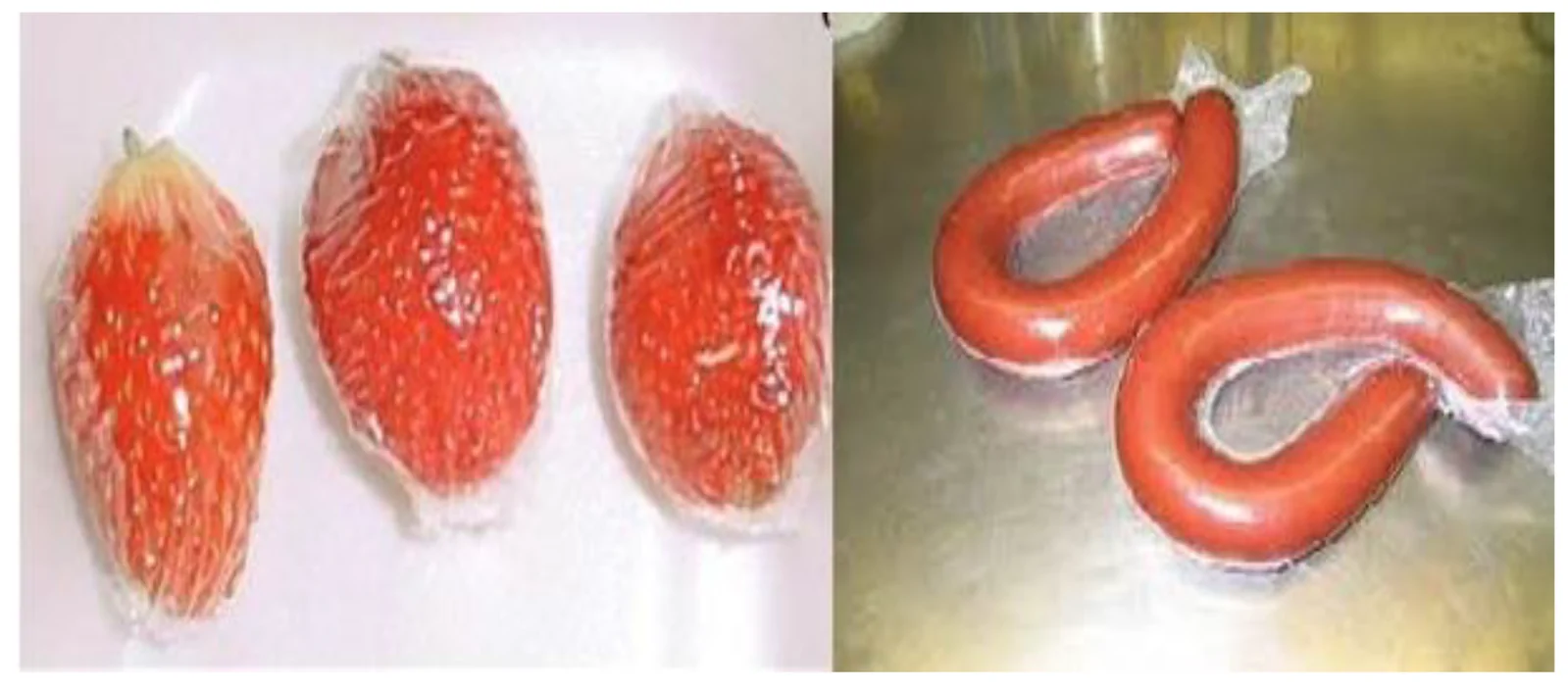
Foods coated with edible film made from polysaccharide material [56].

LBG containing film packaging is generally prepared using the solvent casting method. In this method, LBG is dissolved in heated water at approximately 80 °C for 30 min. Components to be incorporated into the film formulation—such as plasticizers, hydrophobic agents, and other biopolymers—are added to the solution. The resulting mixture is poured onto a flat plate. The material is dried at 20–45 °C for several hours to allow the solvent to evaporate. After the drying process is complete, the resulting films are peeled off the plate [45]. In edible coating applications, the LBG solution is applied to the food surface by dipping, spraying, or brushing and then dried. The coating process involves wetting the food surface with the coating solution and its subsequent adhesion to the surface [5,55]. Figure 5 shows foods coated with film obtained from polysaccharide material [56].

As a packaging material, LBG can exhibit a synergistic effect through hydrogen bonds when combined with other biopolymers such as xanthan, guar gum, carrageenan, tragacanth gum, and pullulan. This improves mechanical and barrier properties. Physical and functional properties critical for food preservation—such as oxygen, carbon dioxide, and water vapor permeability, elongation at break, tensile strength, and flexibility—can be improved in this manner [4,26,38,40,43,45,47,59,60,61]. To increase the quality, durability, and functionality of the resulting film or coating, plasticizers such as polyethylene glycol, glycerol and sorbitol, as well as hydrophobic components like beeswax and fatty acids, can be incorporated [10,39,45,46].

### 5.3. Physicochemical Properties of LBG-Based Films

Plasticizers are utilized in LBG-containing film packaging to reduce brittleness, ensure homogeneity and dispersibility, and improve flexibility [11,62]. In a study investigating the effects of plasticizers on biopolymer films, Mikkonen et al. produced films by adding glycerol and sorbitol at ratios of 20–60% to LBG and guar gum. They reported that reducing the degree of polymerization of guar gum via enzymatic treatment improved the mechanical properties of the films. Furthermore, higher values of elongation at break and tensile strength were determined in galactomannans with lower galactose content. This was attributed to the tighter association of the mannose main chains as the amount of galactose in the side chains decreased [63]. In the same study, the use of glycerol and sorbitol plasticizers provided a more homogeneous and flexible film structure. Increasing the concentration of plasticizers generally led to an increase in elongation at break and a decrease in tensile strength [39,63].

In another study examining the barrier properties of LBG [39], films prepared by adding different plasticizers (glycerol, propylene glycol, sorbitol, and polyethylene glycol 200 (PEG 200)) and hydrophobic materials (stearin and beeswax) were tested. The lowest water vapor permeability was obtained in films containing PEG 200 and sorbitol, whereas the highest water vapor permeability was observed in films with glycerol. The authors reported that PEG 200 was the most suitable among the tested plasticizers as it provided the lowest water vapor permeability, and the maximum concentration for use was 0.6 mL/0.7 g LBG. Since biopolymers are hydrophilic, incorporating hydrophobic substances into the film formulation is a common approach to reduce water vapor permeability. Similarly, in this study, samples with added stearin and beeswax as hydrophobic materials were found to exhibit lower water vapor permeability compared to those without [39].

Martins et al. developed edible films from blends of κ-carrageenan and LBG mixtures. In the study, 30% (*w*/*w*) glycerol was used as a plasticizer, and the two biopolymers were mixed at different ratios: 100/0, 80/20, 60/40, 40/60, 20/80, and 0/100 (*w*/*w*). According to the results obtained, the films with a κ-carrageenan/LBG ratio of 40/60 (*w*/*w*) exhibited the best water vapor barrier and mechanical properties. The combined use of the two biopolymers demonstrated a synergistic effect, enhancing barrier properties and reducing water vapor permeability. Furthermore, improvements in elongation at break (EB) and tensile strength (TS) values were observed compared to the individual use of either biopolymer. This result is significant in terms of overcoming one of the most critical limitations of hydrophilic LBG in packaging production [61].

In another study conducted by Kurt et al., edible films were produced using different ratios of LBG, xanthan gum, and glycerol. The study identified that through the interaction of these two biopolymers, they could be successfully utilized in biodegradable film production. The LBG, xanthan gum, and glycerol concentrations of the optimized film sample were determined as 89.6%, 10.4%, and 20%, respectively [64].

In a study where films were formed using LBG and gum tragacanth, FTIR spectra revealed non-covalent interactions between the molecules of these two biopolymers. Viscosity data also indicated a synergistic effect between them regarding film formation. Additionally, their combined use was reported to decrease surface tension, which is crucial for better spreading of the film formulation on surfaces and forming a more homogeneous structure. Regarding moisture absorption, LBG films absorbed more moisture than gum tragacanth films. The moisture absorption of the blend films was lower than that of LBG films and higher than that of gum tragacanth films. This result was observed regardless of mixing ratios. In terms of water vapor permeability, LBG films exhibited lower permeability compared to gum tragacanth films. In blend films, the addition of LBG reduced the water vapor permeability of gum tragacanth [40].

The studies highlighted above demonstrate that plasticizers enhance the processability of LBG films and that the synergistic interaction of LBG with other biopolymers through non-covalent interactions and tighter chain association exerts a positive effect on film properties. In particular, mechanical strength and water vapor barrier performances can be effectively improved in this manner.

### 5.4. Use of LBG as Coating and Active Packaging in Food Applications

Coating fruits with edible coatings post-harvest is a simple, economical, and environmentally friendly method that can be used as a cooling aid to extend product shelf life and enhance stability during storage. Edible coatings reduce the respiration rate of fruits and control oxidation as well as water vapor permeability. Consequently, fruits can maintain their firmness, original color, and freshness for a longer period [1,8,55].

The gas permeability properties of beeswax–hydrocolloid coatings applied to citrus fruits were compared with commercial coatings [65]. In this study, xanthan, LBG, or guar gum was added to beeswax. No statistically significant difference was observed among the water vapor permeability values of the resulting films. However, the addition of guar gum and LBG to beeswax coatings reduced oxygen and carbon dioxide permeability [65].

In another study involving fruit coating, three different lipid/hydrocolloid coatings, including LBG, were applied to evaluate the post-harvest shelf-life quality of freshly harvested ‘Golden Delicious’ apples [66]. The apples were cold-stored for 8 weeks, and it was reported that gas exchange was reduced and fruit firmness loss was lower in the coated groups compared to the uncoated control group. Furthermore, it was emphasized that using lipids in combination with polysaccharides in the coatings reduced water vapor permeability, and that lipids and carbohydrates exerted a synergistic effect in maintaining post-harvest quality and extending shelf life [66].

The studies highlighted above demonstrate that integrating LBG into composite coating formulations provides a dual functionality for post-harvest fruit preservation. While LBG effectively restricts oxygen and carbon dioxide exchange to slow down respiration, the incorporation of lipids counteracts moisture loss by lowering water vapor permeability. This synergistic interaction between LBG and lipids plays a crucial role in suppressing metabolic degradation, thereby preserving tissue firmness and extending the overall post-harvest shelf life.

The efficacy of LBG in controlling post-harvest decay in mandarins was also investigated. In the study, when the yeasts *Wickerhamomyces anomalus* BS91, *Metschnikowia pulcherrima* MPR3, and *Aureobasidium pullulans* PI1 were used in combination with LBG, yeast cell viability increased, and post-harvest decay caused by *P. digitatum* and *P. italicum* were controlled [67].

Similarly, the LBG-based coating enriched with *W. anomalus* yeast reduced weight loss, maintained firmness, and inhibited green mold formation during the storage of ‘Valencia’ oranges [68]. Furthermore, edible coatings produced from chitosan and LBG enriched with pomegranate peel extract were determined to prevent the growth of *Penicillium digitatum* on oranges [69].

Biopolymer-derived edible films, in addition to providing moisture and gas barrier properties, possess strong potential to serve as effective carrier matrices for antimicrobial and antioxidant agents, nutritional components, colorants, and anti-browning agents. Therefore, they are considered a significant alternative in the development of functional packaging materials [6,10,38]. In a study regarding LBG-based active films [26], the effects of adding daphnetin to a film formulation formed from a blend of sodium alginate and LBG were investigated. According to the results, the addition of daphnetin at low concentrations increased the flexibility of the sodium alginate–LBG film and improved both elongation at break and tensile strength. As the daphnetin content increased, the solubility, gloss, and transparency of the film decreased, whereas moisture permeability increased. The antioxidant capacity and antibacterial activity of the daphnetin-containing films were significantly enhanced compared to the basal film. These results indicate that the combination of daphnetin in the sodium alginate–LBG film provides an active film with antioxidant and antibacterial properties, demonstrating potential for use as a food packaging material [26].

Furthermore, incorporating antimicrobial agents into edible packaging rather than adding them directly to the food matrix ensures a slower and more controlled release of these components into the food. This mechanism enables prolonged preservation of the product [6,10].

Coatings prepared from a blend of LBG and sodium alginate containing different concentrations (0.16; 0.32; 0.64 mg·mL^−1^) of daphnetin as an antimicrobial agent were tested on turbot (*Scophthalmus maximus*) during 18 days of cold storage (4 ± 1 °C) [70]. The results demonstrated that LBG-sodium alginate coatings containing 0.32 mg·mL^−1^ daphnetin significantly reduced the total viable bacterial count, psychrophilic bacteria count, and *Pseudomonas* spp. and H_2_S-producing bacteria counts. In addition, it prevented the formation of undesirable flavor compounds, including total volatile basic nitrogen, trimethylamine, and ATP-related compounds. The daphnetin-containing LBG-sodium alginate coatings also delayed myofibrillar degradation in the turbot samples. These findings reveal that LBG-sodium alginate coatings containing 0.32 mg·mL^−1^ daphnetin represent a potential alternative for preserving the quality of turbot during refrigerated storage [70].

In another study, they reported that LBG/κ-carrageenan/whey protein blend films were reported to be a suitable carrier for the probiotic *Lactobacillus rhamnosus* GG. The authors noted that the viability of *L. rhamnosus* GG was maintained and its losses were minimized during 25 days of storage at 4 and 25 °C for 25 days [71].

The post harvest and food preservation studies summarized above collectively demonstrate that LBG is not merely a passive physical barrier, but an exceptionally versatile delivery vehicle (carrier matrix) for active bio-agents, including biocontrol yeasts, plant extracts, purified polyphenols, and probiotics.

In a study, edible films were produced using whey protein isolate with four different concentrations of LBG and two different heat treatments. The barrier, mechanical, and optical properties, as well as the microstructure, solubility, and moisture absorption behavior of the resulting films, were investigated. The results demonstrated that the interaction between whey protein isolates and LBG and more severe heat treatments yielded stronger, more flexible, and less soluble films with lower permeability to carbon dioxide and oxygen [60]. These findings indicate that thermal processing coupled with protein–polysaccharide interactions effectively reinforces the structural integrity and barrier performance of LBG-based films.

### 5.5. Overall Evaluation

All these results demonstrate that LBG can be successfully utilized in the production of biodegradable packaging. LBG possesses a structure compatible with other biopolymers and can be considered a suitable carrier matrix for active components such as antimicrobial agents and phenolic compounds that enhance nutritional value. Due to its polar nature, LBG exhibits strong barrier properties against oxygen and carbon dioxide. However, owing to its hydrophilic nature, it is a material with high water vapor permeability. Furthermore, its mechanical strength and flexibility are weaker compared to conventional plastic packaging materials. Nonetheless, these limitations can be overcome by incorporating hydrophobic compounds, plasticizers, or other polymers into the film formulation.

## 6. Use of Pectin in Edible Packaging Production

**Figure 6:**
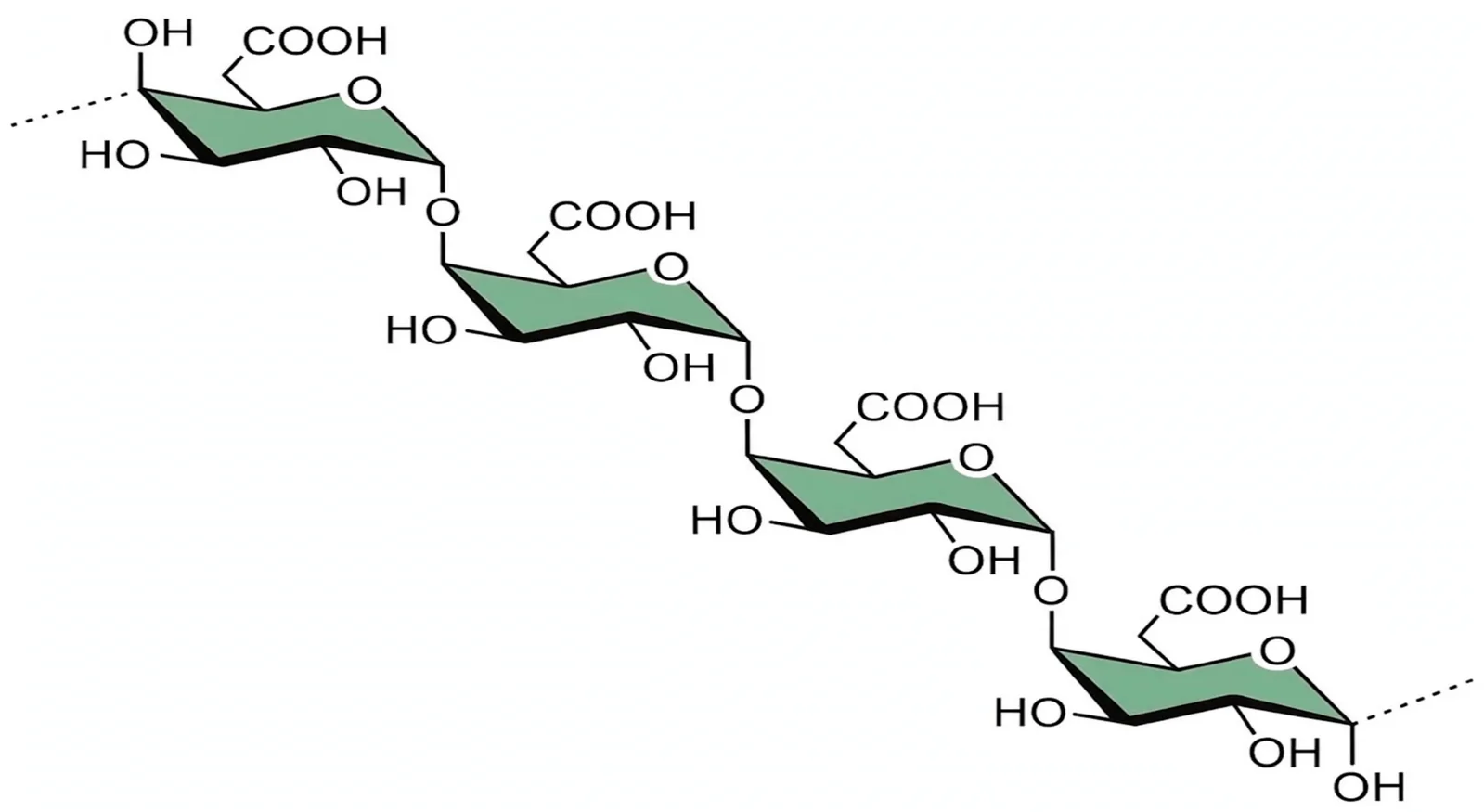
Schematic representation of the structure of pectin.

In addition to its seed gum (LBG), which is widely known in the industry, the carob pod contains high-quality pectin in its pulp. Carob pectin, like other polysaccharide biopolymers found in carob, has the potential to form an edible matrix as an alternative to synthetic polymers in biodegradable packaging technology. Nevertheless, the pulp, which constitutes the remaining 90% of the pod and is largely regarded as a by-product, is increasingly being utilized in the food industry. The fruit pulp contains high amounts of sugars; insoluble fibers such as cellulose, hemicellulose, and lignin; phenolic compounds known for their antioxidant properties (among which carob fruit is the primary natural source of D-pinitol); minerals such as Ca, P, and Mg; and pectin [34,72,73]. Pectin is a polysaccharide composed of galacturonic acid units linked by α-(1 → 4) bonds and is abundant in plant cell walls (Figure 6). It is used in the food industry as a gelling agent, thickener, stabilizer, and hydrocolloid agent. Owing to these properties, it is widely utilized in the production of foods such as jams, juices, desserts, and dairy products [74,75,76]. Pectin is one of the most frequently used natural polymers in the production of edible packaging [10]. Recently, numerous studies have been conducted wherein pectins obtained from different sources were incorporated into biodegradable films and coatings for the preservation of various foods, such as fruits, vegetables, and meat products [77,78,79,80,81,82,83].

### 6.1. Film and Coating Production

Pectin-based films are generally obtained by the solvent casting method. The casting method is performed by pouring the film-forming solution thinly onto a non-stick plate or mold, drying it, and peeling the dried film from the mold [4,62,73,84]. As for coatings used particularly for the preservation of fresh fruits and vegetables, they can be applied through dipping, spraying, or brushing methods [85]. Pectins, like other polysaccharide biopolymers, are compounds with low gas permeability but high water vapor permeability. In edible packaging production, their combined use with different polymers makes it possible to improve their negative characteristics regarding film and coating production. The excellent gelling ability and amorphous structure of pectins also facilitate the incorporation of additives into the film [62,73,74].

### 6.2. Source and Extraction

Apple pomace and citrus peel are the primary sources for commercial pectin production. However, since pectin is one of the fundamental components of the plant cell wall, it can be obtained from various sources [62,73]. Carob pod also contains a significant amount of pectin [86]. The pectin content in carob was determined as 4.9 g/100 g dry matter [87]. The chemical composition of pectin found in carob pulp can vary significantly depending on the cultivar, cultivation conditions, and agricultural practices [34]. Pectin extraction can be performed through conventional extraction using organic and inorganic acids, or via non-conventional methods such as enzyme-assisted, microwave-assisted, ultrasound-assisted, and subcritical water extraction. The conventional method is based on destroying the plant cell wall using a strong acid, such as hydrochloric acid or sulfuric acid, to release the pectin. The extraction process is generally carried out at a pH of around 1.5, at temperatures of 70–90 °C, and for a duration of 90 min. A dissolved pectin extract is obtained through the combined effects of temperature and acid. Following extraction, the extract is passed through a filter to remove insoluble residues. Subsequently, pectin is precipitated using alcohols such as ethanol or isopropanol. Washing is then performed several times with alcohol to remove impurities. After the washing step, drying and milling processes are carried out to yield pectin in powder form [75,76].

### 6.3. Structural Properties and Gelling

Pectins are divided into two main categories according to the degree of esterification of their carboxyl groups. Pectins in which more than half of the carboxyl groups are in the methyl ester form are termed high-methoxyl pectin (HM pectin), whereas those with less than 50% esterification are designated as low-methoxyl pectin (LM pectin). This characteristic is decisive for the gelation mechanism of pectins [34,62,75,85]. For HM pectins to form a gel, a high sugar content and high acidity are required. Due to the high proportion of methoxyl groups, more sugar is needed to bind water. Therefore, they are utilized to form a firm gel structure in high-sugar foods such as jams, jellies, marmalades, and confectionery. LM pectins, on the other hand, form gels in the presence of calcium ions. The gelling mechanism of LM pectins is explained by the “egg-box model.” Free carboxyl groups along the chains form ionic bridges with Ca^2+^ ions creating a three-dimensional gel network between the chains. Owing to these properties, they are preferred in low-sugar or sugar-free products where high acidity is not desired, such as yogurt, dairy beverages, fruit drinks, and canned fruits [34,62,73,75,85]. The structural characteristics of carob pectin are influenced by numerous factors such as cultivar, degree of maturity, extraction method, and molecular weight [34]. In a study, the degree of esterification of pectin, across six different carob cultivars, was determined to be in the range of 45–70%, with an average value of 58.3%. Based on these results, the examined carobs were found to exhibit predominantly HM characteristics. The same study emphasized that pectins present in carob pulp have the potential to form edible films due to their HM properties [34]. Furthermore, the carob pectin fraction also contains antioxidant phenolic compounds [88]. Therefore, films obtained from carob pectin have the potential to be used as active packaging materials that delay oxidative degradation in foods.

### 6.4. Overall Evaluation

No studies have been found in the literature regarding the direct use of pectins extracted from carob pulp in the production of edible packaging. However, considering the potential of pectins from various fruit peels and pomaces in film and coating production, carob pectin is anticipated to hold significant potential for use in edible packaging. In particular, phenolic compounds bound to carob pectin could impart antioxidant properties to this polymer. This enables carob pectin-based films to be considered as active packaging materials. Moreover, carob-based composite edible films could also be developed by combining LBG obtained from carob seeds with other polysaccharides present in carob pulp, such as cellulose, pectin and hemicellulose. This approach is industrially valuable for minimizing waste through the valorization of both pulp and seeds.

## 7. Use of Cellulose in Edible Packaging Production

**Figure 7:**
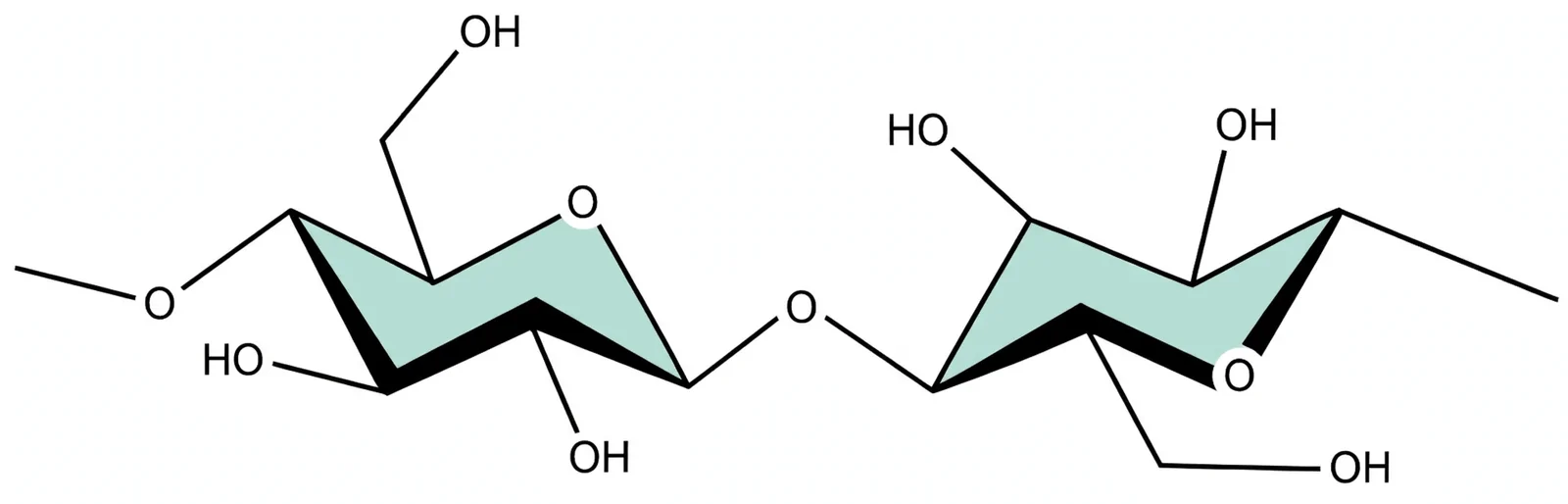
Schematic representation of the chemical structure of cellulose.

Cellulose is the most abundant biopolymer in nature, produced by plants, microorganisms, algae, and fungi [89,90]. It is the structural polysaccharide of the cell wall in plants and provides mechanical strength to the plant [88]. Cellulose is a linear polysaccharide composed of D-glucose monomers linked by β-(1 → 4) glycosidic bonds (Figure 7) [86]. Due to strong hydrogen bonds and crystalline regions between its chains, cellulose is insoluble in water and most organic solvents [56,90]. However, the free hydroxyl groups in glucose units facilitate the production of water-soluble derivatives by offering reactive sites for chemical modification. The production of water-soluble derivatives with different properties through physical and chemical modification gives cellulose versatility, thus enabling it to have a wide range of uses in industry for many different purposes [91]. Cellulose is widely used in industries such as pharmaceuticals, textiles, paper, and food, and it is primarily sourced from cotton fibers and wood pulp [56,92]. However, in recent years, cellulose has also been increasingly obtained from alternative sources, such as herbaceous plants. In particular, agricultural and food industry by-products contain high amounts of cellulose, and their use for different purposes in industry is important in terms of the sustainability of production and the protection of the environment [89]. Furthermore, cellulose can be obtained as an exopolysaccharide from microorganisms, i.e., bacterial cellulose, which is notable for its high purity, being free of hemicellulose and lignin [93].

### 7.1. Cellulose and Its Derivatives

The stable main chain of cellulose, formed by β(1 → 4) glycosidic bonds, allows for the addition of numerous functional groups without undergoing structural degradation during derivatization reactions [92]. In this context, derivatives of cellulose can be obtained through physical and chemical modifications to improve properties such as solubility and thermoplastic behavior. Physical modification is carried out using techniques such as mechanical, thermal, electrical, gamma irradiation, and UV light. Chemical modifications include techniques such as hydrolysis, esterification, acetylation, and etherification [91]. Cellulose has a wide range of industrial applications due to its diverse derivatives, including carboxymethyl cellulose (CMC), methylcellulose (MC), cellulose acetate (CA), cellulose sulfate (CS), ethylcellulose (EC), hydroxyethyl cellulose (HEC), hydroxypropyl cellulose (HPC), and hydroxypropyl methylcellulose (HPMC). Cellulose is a widely used material in edible packaging production and offers significant potential for advancing this technology, owing to its versatile properties, biodegradability, biocompatibility, high thermal stability, effective UV protection, easy availability, lightweight nature, and sustainability. However, cellulose-based films exhibit certain limitations for packaging applications, such as generally poor water vapor barrier properties due to their hydrophilic nature [91,92,94,95]. Furthermore, pure cellulose lacks antimicrobial and antioxidant properties, which limits its ability to actively protect food products [96]. However, its excellent carrier and matrix properties enable the incorporation of antioxidant and antimicrobial agents into the film or coating formulation [94]. In this context, the effect of 0.5% CMC biodegradable coatings enriched with a bacteriocin purified from *Bacillus methylotrophicus* BM47 on extending the shelf life of fresh strawberries was investigated. After 16 days of storage at 4 °C and 75% relative humidity, the coating led to a significant reduction in weight loss in coated strawberries compared to uncoated fruits, and also delayed both decay and the loss of antioxidant activity [97]. In another study utilizing a different cellulose derivative, pink pepper essential oil was incorporated into CA films. This incorporation exhibited inhibitory effects against *Staphylococcus aureus*, *Listeria monocytogenes*, *Escherichia coli*, and *Salmonella typhimurium* in sliced mozzarella cheese [98]. In a study on strawberries, Liu et al. investigated the effects of composite coatings based on HEC and sodium alginate enriched with asparagus by-product extract. The coatings exhibited antifungal activity against *Penicillium italicum*, delayed color changes in the fruit, and preserved total phenolic and flavonoid contents. These results indicate that the coatings have the potential to extend the postharvest shelf life of strawberries [99]. The studies highlighted above demonstrate that incorporating bioactive compounds into cellulose derivative matrices effectively overcomes the functional limitations of pure cellulose, transforming passive barrier coatings into active systems that suppress microbial decay and preserve fruit quality.

### 7.2. Cellulose Extraction

It can be performed by different methods such as conventional extraction, ultrasound-assisted extraction, and microwave-assisted extraction [100]. In conventional cellulose extraction from high-sugar fruits such as carob, sugars are first removed from the solution to prevent caramelization and adhesion to cellulose under alkaline conditions. For this purpose, the dried and ground pomace is boiled in distilled water at 80–90 °C for 1–2 h and then filtered. Subsequently, it is treated with 2–5% dilute alkali (NaOH) at 80 °C for 2–4 h to dissolve and remove hemicellulose and pectin present in the cell wall. The fibers obtained after the alkaline treatment appear brown because lignin is still present in the structure. Lignin is then removed by oxidative bleaching using NaClO_2_ or H_2_O_2_. The resulting bleached fibers consist of pure cellulose. Finally, the fibers are washed with distilled water until neutral pH is reached, dried in an oven at 60 °C, and ground prior to storage [34,86,100,101,102].

### 7.3. Film and Coating Production

In the production of edible films, the complex hydrogen bonds within the structure of cellulose act as a factor that hinders its dissolution and processing in water. To overcome this challenge, researchers have developed novel solvents. These are used or utilized derivatives obtained through chemical modifications (such as CMC and HPMC) [90]. Solvent casting is widely preferred for the production of cellulose and cellulose-derivative films [95,103]. As for coatings, dipping, spraying, or brushing are frequently employed methods [95,104]. After separating the seeds of carob fruits, the sugars present in the pomace are generally extracted to produce molasses or syrup. The pulp or by product remaining after the extraction of sugars contains a significant amount of cellulose [86]. In a study, following the removal of seeds from various carob fruit varieties, the cellulose content in fresh carob pulp has been reported to range between 5.6 and 9.3 g per 100 g [34]. In another study, it was reported that the remaining pulp of carob after seed separation contained 7.64% cellulose [105]. These values indicate that carob pod is a sustainable source of cellulose. In a study, cellulose-rich extracts were produced from pulps obtained from various carob varieties. The functional properties of these extracts—such as antioxidant activity and total phenolic content—along with parameters critical for edible packaging applications, including swelling capacity, water-holding capacity, and oil adsorption, were investigated. The findings indicated that carob pulp, an agricultural by-product, is a rich source of cellulose and possesses high potential for food gel and edible packaging production due to its structural suitability for forming compact and firm gels. Furthermore, due to the variability observed among different carob varieties, the study highlighted the importance of cultivar selection for new applications [34]. In a related study by Capella et al., cellulose-rich fractions derived from carob pulp were combined with potato peel flour to formulate 3D-printed food gels. In the study, the functional properties of cellulose-rich fractions that influence gelling behavior, such as swelling capacity, water-holding capacity, and oil-holding capacity, were determined. These properties are also directly related to edible film performance, as swelling and water/oil-holding capacities are key parameters that determine the barrier, mechanical, and structural integrity of the film [86]. The findings indicate that carob pulp, an agricultural byproduct, can be considered a source of cellulose, and that cellulose-based structures can be used as matrix-forming materials in food systems.

### 7.4. Overall Evaluation

The results mentioned above show that cellulose isolated from carob pulp can be a potential raw material for edible film and coating applications. The high water-holding and gelling properties of the obtained cellulose-rich fractions indicate that they can be used as matrices to prevent moisture loss in applications such as fresh fruit and vegetable coatings. However, due to the hydrophilic nature of cellulose, the films are expected to exhibit high water vapor permeability; therefore, combination with hydrophobic components is recommended to improve their barrier properties. Some studies providing information on the potential use of carob biopolymers in the production of edible packaging are shown in Table 2.

## 8. Conclusions

The use of edible films and coatings as food packaging has significant potential. They help reduce environmental pollution due to their biodegradability, offer high safety in food-packaging interactions because they are biocompatible, and provide additional health benefits to consumers since they can be consumed with the food. However, for the commercial use of edible packaging, properties such as production costs, mechanical strength, and gas and water vapor permeability must be carefully evaluated.

This study examined the significant potential of carob-derived biopolymers, specifically LBG from the seeds and pectin and cellulose from the pulp, for developing edible packaging matrices. Overall, the presence of distinct biopolymers within a single plant source makes carob a remarkable raw material for sustainable packaging production. Among these, LBG exhibits excellent compatibility with other biopolymers and serves as a suitable carrier matrix for active ingredients, such as antimicrobial agents and health-promoting phenolic compounds. Due to its polar structure, LBG provides good barrier properties against oxygen and carbon dioxide. Although its hydrophilic nature results in high water vapor permeability and its mechanical strength remains lower than that of conventional plastics, these limitations can be effectively mitigated by incorporating hydrophobic compounds, plasticizers, or other polymers.

While studies on carob LBG are relatively abundant in the literature, research directly focusing on carob pulp-derived pectin and cellulose for edible film production remains quite limited. Notably, no previous study has addressed the direct production of edible packaging from carob pulp pectin. However, considering the proven capacity of pectins obtained from different fruit peels and pomaces for film and coating production, it is anticipated that carob pectin also has significant potential for use in edible packaging production. In particular, the phenolic compounds bound to carob pectin can impart antioxidant functionality to the polymer during packaging production, allowing carob pectin-based films to be evaluated as active packaging materials. Cellulose-based structures are widely used as matrix-forming materials in the production of edible films and coatings. Therefore, cellulose isolated from carob pulp is also considered a potential raw material for edible film and coating applications. To successfully integrate carob biopolymers into the industrial packaging sector, further research is required. This includes optimizing the extraction processes of pectin and cellulose from the carob pulp, investigating the film-forming performances of these biopolymers in various synergistic combinations, and conducting shelf-life and sensory evaluations of the developed packaging on actual food systems.

From a future perspective, the translation of carob-derived biopolymers, including LBG from the seeds and pectin and cellulose from the pulp, into commercial edible packaging will depend not only on material performance but also on consumption-related aspects. While all three biopolymers are plant-based and generally considered safe, with LBG being recognized as GRAS and approved as food additive E410 in the EU, their use as edible films raises new considerations regarding sensory properties, digestibility, and consumer acceptance. The characteristic sweet, cocoa-like flavor of carob pulp may be advantageous for certain food applications, while the incorporation of active agents into these matrices could alter taste, aroma, and mouthfeel. Therefore, future studies should complement shelf-life assessments with in vitro digestion studies and consumer acceptance tests to evaluate the willingness to consume packaging derived from different carob biopolymers in real food systems.

In conclusion, carob-derived biopolymers have the potential to offer an environmentally friendly alternative to conventional plastic packaging. Future research and development efforts in this field will contribute to the circular economy by both valorizing agricultural by-products and reducing food waste.

## Figures and Tables

**Table 1:** A comparison of the mechanical and barrier properties of conventional LDPE and biodegradable polymer-based films.

Properties	Conventional (Pure LDPE) [23]	Native Corn Starch [24]	Caseinate + Carboxymethyl Chitosan + Blackseed Oil [25]	Locust Bean Gum + Sodium Alginate [26]
Tensile strength (MPa)	6.13	6.00	12.5	7.52
Elongation (%)	121.39	20	90.5	8.9
WVP (g·mm/m^2^·h·kPa)	0.58	6	0.56	11.20
OTR (cc·m^2^/day)	353	4	-	-

**Table 2:** Overview of Carob-derived Biopolymers for Edible Film and Coating Applications.

Components	Formulation/Product	Evidence Level for Film and Coating Application	Key Properties Reported	References
LBG + Sunflower Protein Film	LBG (0.10–0.75%) + Sunflower Protein; Solvent Casting Method	Direct evidence	Compared to pristine sunflower protein film, incorporation of Locust Bean Gum increased tensile strength and stretchability. Limitation: Slightly increased Water Vapor and Oxygen Permeability	[106]
LBG Coating	LBG + PEG 200, dipping on sausage	Direct evidence	Reduced moisture loss and extended shelf life at 5 °C. PEG 200 showed lowest moisture loss.	[107]
LBG + Xanthan Gum Film	LBG + Xanthan Gum + Glycerol; Solvent Casting Method	Direct evidence	LBG and Xanthan Gum were successfully used together for edible film production	[64]
Carob Pulp Cellulose-Rich Extracts	Cellulose-rich extracts from carob pulp; Gel Formation	Indirect/ Preliminary	Cellulose-rich fractions contain cellulose as the main component, along with pectins and hemicelluloses. Extracts showed high antioxidant activity and phenolic content. They exhibited good swelling capacity, water-holding capacity, and oil adsorption. Carob pulp is structurally suitable for forming compact and firm gels with high potential for edible packaging applications	[34]
Carob Pulp Cellulose-rich Fractions + Potato Peel Flour	Cellulose-rich fractions from carob pulp combined with potato peel flour; 3D-printed Food Gels	Indirect/ Preliminary	The swelling capacity, water-holding capacity, and oil-holding capacity of cellulose-rich fractions of carob pod were determined as properties that influence gelling behavior. These properties are key parameters as they determine the barrier, mechanical, and structural integrity of the film. The findings indicate that carob pulp cellulose-rich fractions can be used as matrix-forming materials in food systems	[86]

## Data Availability

No new data were created or analyzed in this study. Data sharing is not applicable to this article.
